# Immunobullous Transformation of Protracted Psoriasis: A Diagnostic Dilemma

**DOI:** 10.7759/cureus.37639

**Published:** 2023-04-16

**Authors:** Kirtanya Ramachandran, Rashmi Kallabbe Shridhar, Shriram Vaidya, Leen Heis, John Mee

**Affiliations:** 1 Geriatrics, Queens Hospital Center, Romford, GBR; 2 Intensive Therapy Unit, King George Hospital, Ilford, GBR; 3 Dermatology, Queens Hospital Center, Romford, GBR; 4 Histopathology, St Thomas' Hospital, London, GBR

**Keywords:** dysphagia, autoimmune, treatment, psoriasis, pemphigus vulgaris

## Abstract

Pemphigus vulgaris is a rare autoimmune disorder, characterised by the development of blistering lesions in the skin and mucosal surfaces throughout the body. It is often misdiagnosed or missed completely in many patients, prolonging their suffering for many years, as it has the ability to mimic an array of other skin diseases.

Many studies have concluded that there is a strong association between pemphigus vulgaris and psoriasis, though the exact mechanism is not clearly understood. We present the case of a 77-year-old gentleman on long-term treatment for psoriasis with ultraviolet B phototherapy, steroids, and many other topical treatments who eventually went on to develop pemphigus vulgaris.

## Introduction

Pemphigus vulgaris is an autoimmune intraepithelial blistering disease mediated by circulating immunoglobulin G (IgG) autoantibodies against anti-desmoglein 3 extracellular domains of cell membrane proteins of keratinocytes. This potentially life-threatening disease with 5-15% mortality is characterised by acantholysis and loss of cell-cell adhesion between keratinocytes. Immune-mediated skin diseases like pemphigus have been associated with other autoimmune disorders and have the potential to induce occurrence of another immune-mediated skin disease [1]. Many of the treatment modalities including but not limited to ultraviolet B (UVB) therapy and steroids are linked to the development of immune-mediated skin diseases as well [2]. Our case report elaborates on the risk factors and the mechanisms through which they predispose to the evolution of pemphigus vulgaris with special emphasis on the contribution of another coexisting immune-mediated disorder like psoriasis.

## Case presentation

We discuss a case of a 77-year-old gentleman with eczema (atopic eczema) and psoriasis (chronic plaque psoriasis) whose skin disease was poorly responsive to therapies necessitating protracted treatment with Psoralen ultraviolet A (PUVA), methotrexate and retinoid treatment for many months. He also had a background of prostate cancer, hypertension, ischemic heart disease and mixed hyperlipidemia. He had undergone transurethral resection of the prostate for prostatic cancer and was regularly followed up with interval monitoring of the prostate-specific antigen level to rule out recurrence. There was no family history of any chronic skin pathology. His regular medications included amlodipine, ramipril for hypertension and atorvastatin for hypercholesterolemia for 15 years and calcium supplements.

**Figure 1:**
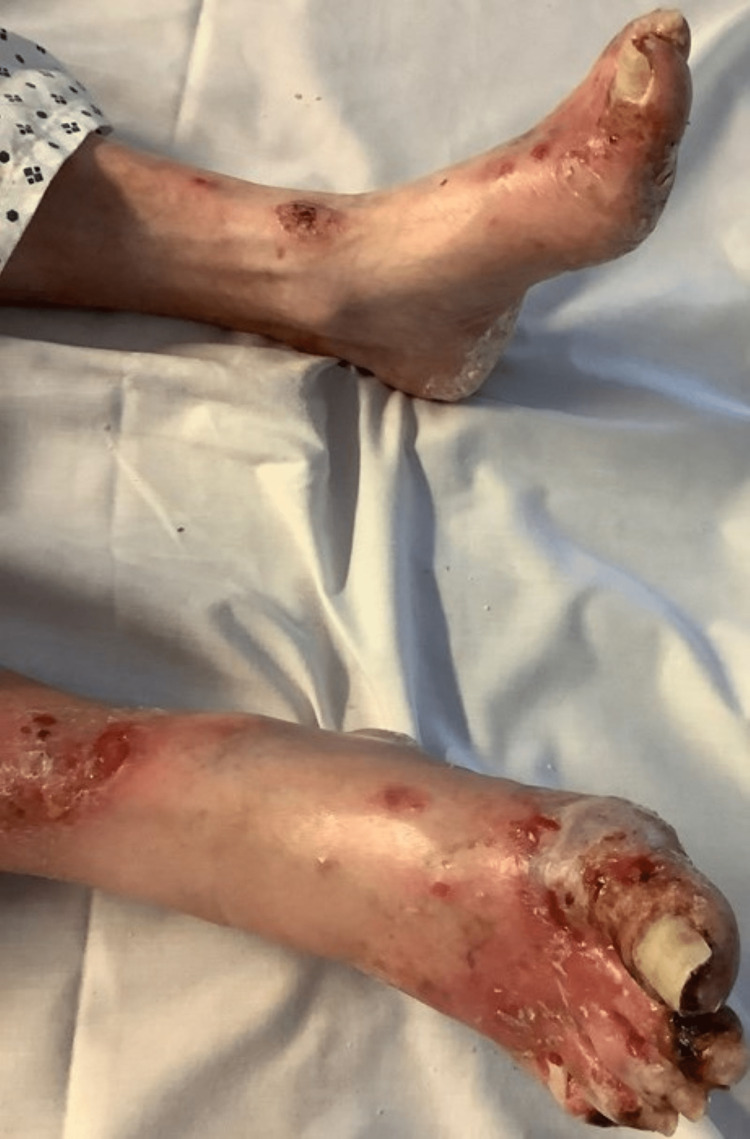
Multiple blisters and erosions noted in the feet that were severely affected.

He was admitted with a gradual onset of generalised blistering itchy rash evolving over a duration of three to five days which began from toes. He also suffered from a gradual onset of dysphagia and odynophagia for the past few days for both liquids and solids. He did not complain of pain, fever, nausea or vomiting. Examination of the rest of the organ systems was unremarkable, and vital signs were stable. On examination, he had flaccid blistering weeping skin lesions all over his body with a watery discharge with both feet and medial thighs being more severely affected (Figure 1). During examination of the blisters, we noticed that applying lateral pressure on the blisters near the border resulted in slipping away of top layers from the lower layers i.e. positive Nikolsky sign. Oral mucosa showed painful erosions, superficial blisters and small ulcers.

Routine blood tests were unremarkable except for a raised C-reactive protein of 90mg/L. Viral markers including human immunodeficiency virus, hepatitis B virus, hepatitis C virus, hepatitis A virus, and Cytomegalovirus were negative. Esophagogastroduodenoscopy showed friable mucosa and oesophageal ulceration. He was started on intravenous (IV) fluids and IV broad-spectrum antibiotics with a provisional diagnosis of exacerbation of eczema with secondary infection. He developed oral candidiasis during his stay in the hospital which was treated with a course of enteral fluconazole for seven days.

**Figure 2:**
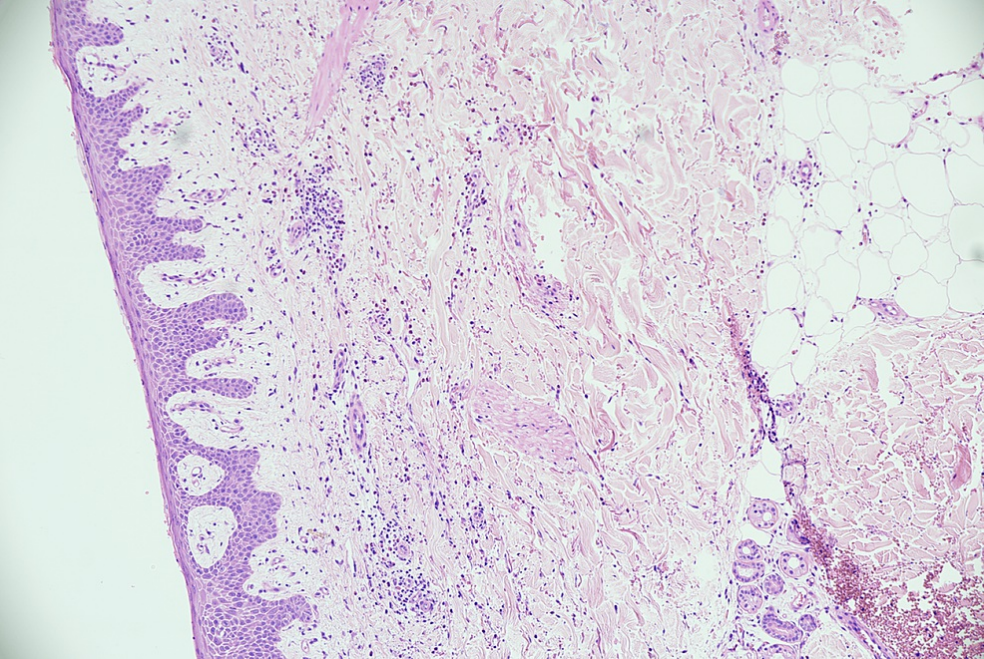
Intraepidermal suprabasal blister without keratinocyte necrosis, giving a “row of tombstones” appearance. Few acantholytic keratinocytes are also seen within the blister cavity with perivascular inflammatory infiltrates and a moderate amount of eosinophils.

**Figure 3:**
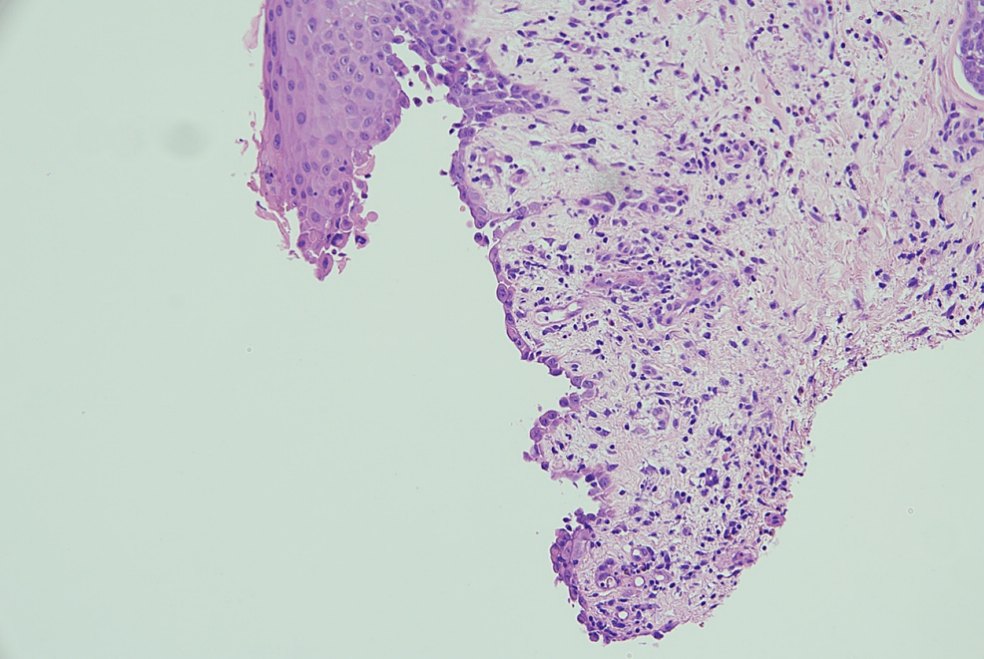
Features are of spongiotic dermatitis. Spongiosis within the epidermis and irregular elongation of rete ridges can be seen.

**Figure 4:**
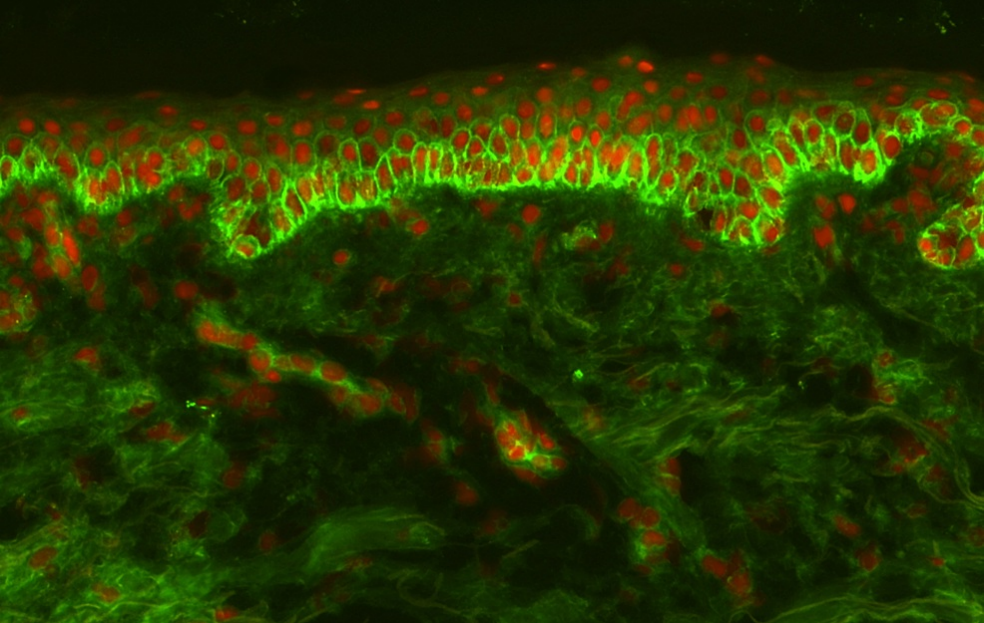
Direct immunofluorescence image showing intercellular deposition of complement 3 in the basal layers of the epithelium consistent with pemphigus.

It was observed that the oral mucosal lesions are not characteristically associated with eczema or psoriasis; hence, work up for alternate diagnosis (skin biopsy) was considered. Microscopy of skin biopsy specimens from the blisters in the medial thigh showed suprabasal acantholysis with tombstone appearance of the basal keratinocytes along with acantholytic keratinocytes (Figure 2). Features of spongiotic dermatitis were also visualised in the basal layers of the epithelium (Figure 3). The superficial dermis showed perivascular lymphocytic infiltrate rich in eosinophils. Direct immunofluorescence revealed extensive intercellular deposition of IgG and complement 3 in the epithelium (Figure 4). These findings were consistent with and characteristic of pemphigus vulgaris. 

He was started on oral prednisolone (60mg once a day) in keeping with the diagnosis of pemphigus vulgaris. The erosions in his thigh and feet were dressed with a special dressing impregnated with neutral triglycerides after completing the thrice weekly potassium permanganate soak for affected areas. Chlorhexidine mouthwash along with a combination mouthwash containing doxycycline, nystatin and betamethasone was used for oral care. Steroid creams betamethasone 0.1% and clobetasol propionate 0.05% were applied for the rest of his body except for the feet, face and genitalia. Liquid paraffin-containing barrier cream was also prescribed, to be applied over the affected areas.

The patient’s skin lesions healed very well to his satisfaction. Hence, he was discharged with a planned review in a dermatology clinic in two weeks. He was continued on prednisolone 30mg/day with instruction to taper further based on the progress of the resolution of skin lesions. Steroid-sparing agent mycophenolate mofetil 500mg twice a day, as the preferred second-line treatment after oral prednisolone, was also prescribed with instruction to gradually increase the dose to 1 g twice a day. Azathioprine was contemplated as another potential treatment. However, given the background of prostate cancer and the drug's potential to increase risk of development of malignancy, mycophenolate mofetil was considered more appropriate. 

During his follow-up visit after two weeks, he had done remarkably well with only minimal post-inflammatory hyperpigmentation at the site of previous blisters, no new blisters and only mild discomfort while eating. He developed onychomadesis and was reassured of likely resolution in 6-9 months. Renal function tests were requested since the patient was on mycophenolate mofetil and were normal. 

## Discussion

Generalised immunobullous disorders can be of many types. Differential diagnoses of flaccid and sloughing bullae (as in this case) include Stevens-Johnson syndrome/toxic epidermal necrolysis, pemphigus vulgaris, pemphigus foliaceus (PF) and paraneoplastic pemphigus (PNP) [3].

Several cases of development of bullous skin diseases in patients with psoriasis and eczema have been reported [4]. Bullous pemphigoid is the most frequent association observed while pemphigus-like disorder is less frequently described [3]. Development of pemphigus after treatment of psoriatic lesions with ultraviolet light, steroids, or immunosuppressants has been reported [5]. Proposed mechanisms for such bullous transformation of a scaly skin disorder include antigen modification, altered regulation of T-cell activity and plasminogen activator abnormalities [6].

As per the meta-analysis conducted by Phan et al., pemphigus was more common in patients with psoriasis than in controls (OR 2.64, 95% CI 1.24-5.59, P=0.01) [1]. In another meta-analysis conducted by Kridin et al. in 2019, the overall pooled multivariate OR for psoriasis in patients with pemphigus was significantly increased and estimated at 3.5 (95% CI, 1.6-7.6) demonstrating evidence of association between these two distinct classes of skin lesions [7].

There are noted associations between PUVA/ UVB therapy and the onset of immunobullous disorders during or shortly after the treatment [8]. PUVA/ UVB therapy was administered in our patient, however, many years back making it an unlikely trigger for transformation of psoriasis into Pemphigus.

A couple of case reports have highlighted drugs such as angiotensin-converting enzyme (ACE) inhibitors playing a role in the pathogenesis of pemphigus. Studies have shown that the serum ACE level was considerably lower in patients with pemphigus vulgaris compared to the control group suggesting an association with pemphigus vulgaris especially among male patients [9]. Studies have also shown that the mean duration between commencement of ACEIs and development of pemphigus is at least 154.27 days [10]. The patient in discussion was on ramipril for a much longer duration, and no such temporal association between starting ramipril and bullous transformation could be established.

Drugs with potential to induce transformation into pemphigus can be categorised into thiol and non-thiol groups. Thiol drugs include captopril, penicillamine and enalapril and are postulated to induce acantholysis through biochemical mechanisms without antibody formation [11]. Non-thiol drugs include penicillins, cephalosporins, antihypertensive agents and piroxicam. A difference in the chemical structure between different drugs within the ACE inhibitor class may be the reason why few drugs are associated with pemphigus while others are not. The presence of a sulphur moiety in thiol drugs could potentially explain this occurrence. The importance of reviewing the medications to identify possible triggers in every case of newly diagnosed or exacerbated pemphigus cannot be overemphasised. Discontinuing any of the culprit medications would reduce the requirement for immunosuppressants [12].

Another interesting facet of this case report is the history of prostate cancer for which the patient had undergone surgical intervention a few years ago. Literature shows that PNP is more commonly associated with neoplasms like Castleman tumour, non-Hodgkin lymphoma, follicular dendritic cell sarcoma and chronic lymphocytic leukaemia [13]. However, the evidence to suggest association between prostate cancer and PNP is limited [14]. It was also noted that PNP is characterised by intact cutaneous blisters and the histologic picture of supra-basilar acantholysis and individual keratinocyte necrosis with eosinophils being rarely seen unlike in our case where there were eosinophil-rich clusters seen in superficial dermis in skin biopsy. Therefore, the possibility of PNP was considered unlikely.

PF was an important differential considered. However, acantholysis observed in the basal layer of epithelium rather than the upper epidermis within or adjacent to the granular layer during direct immunofluorescence analysis of skin biopsy specimens was typical of pemphigus vulgaris.

Impaired suppression of the humoral immune system leads to production of autoantibodies against the skin antigens in psoriasis. This in turn causes perturbation of the epidermal permeability barrier. This Is the primary pathophysiologic mechanism for many skin diseases including pemphigus. It has also been reported that the plasminogen activator which is increasingly present in the psoriatic lesions has the capacity to induce acantholysis in pemphigus [15]. The protracted impairment in the dermal barrier function in our patient who had a background of psoriasis for many years could be the potential triggering factor for the transformation into pemphigus.

## Conclusions

Although several cases of bullous diseases associated with psoriasis and eczema have been reported in the literature, pemphigus-related disorders are less frequently described. Proposed mechanisms include antigen modification, altered regulation of T-cell activity and plasminogen activator abnormalities; however, they are still areas of active research. A number of trigger factors can contribute to the transformation of a scaly skin disorder like psoriasis into an immunobullous disorder like pemphigus vulgaris. It is vital to look for modifiable triggers like steroidal therapy, UVB treatment, immunosuppressants, ACE inhibitors and paraneoplastic syndrome. Triggers can be very elusive and unidentifiable many times. Undiagnosed pemphigus vulgaris is associated with higher mortality and morbidity; hence, a high index of suspicion is warranted to diagnose such immunobullous transformation. In spite of frequent idiopathic nature of such transformation, prompt diagnosis and appropriate modification in therapy are likely to lead to a satisfactory outcome.
